# THE LINK BETWEEN EDUCATION WITHIN SOCIAL NETWORKS AND VOLUNTEERING

**DOI:** 10.1093/geroni/igae098.0600

**Published:** 2024-12-31

**Authors:** Noah Webster

**Affiliations:** University of Michigan, Ann Arbor, Michigan, United States

## Abstract

Volunteering serves as a critical source of social integration in later life and also is a well-known health promoting activity. It is therefore important to continue exploring unidentified barriers and facilitators of volunteering. Both higher education and social networks (e.g., decreasing network proximity) have been linked with volunteering. No research though has explored how educational resources embedded in older adults’ social networks may facilitate this activity. In this study, we examine the association between average social network education and odds of volunteering among older adults. Respondents age 60 and older who participated in Wave 3 (2015) of the longitudinal Social Relations Study based in Metro Detroit were selected for analysis (n=342). During interviews, when asked to identify social network members, respondents were also asked to report the education of up to the first 10. Forty-one percent of respondents reported volunteering in the past year. Controlling for respondents’ own education as well as other demographic characteristics (age, gender, marital status), higher average social network education was significantly associated with greater odds of volunteering. In a regression model without average social network education, individual education was significantly associated with greater odds of volunteering, but this effect was no longer significant after network education was added to the model. Findings highlight that educational resources, often viewed as an indicator of macro-level socio-economic status, when embedded in social networks plays a greater role in facilitating volunteering than at the individual-level. This suggests a potential new pathway for intervention to increase volunteering in later life.

